# HIV-Helminth Co-Infections and Immune Checkpoints: Implications for Cancer Risk in South Africa

**DOI:** 10.3390/v17030451

**Published:** 2025-03-20

**Authors:** Botle Precious Damane, Thanyani Victor Mulaudzi, Sayed Shakeel Kader, Pragalathan Naidoo, Zodwa Dlamini, Zilungile Lynette Mkhize-Kwitshana

**Affiliations:** 1 Department of Surgery, Steve Biko Academic Hospital, University of Pretoria, Hatfield 0028, South Africa; botle.damane@up.ac.za (B.P.D.); thanyani.mulaudzi@up.ac.za (T.V.M.); 2 Department of Medical Microbiology, College of Health Sciences, School of Laboratory Medicine & Medical Sciences, Nelson R. Mandela School of Medicine, University of KwaZulu-Natal, Durban 4041, South Africa; 3 Department of Surgery, University of KwaZulu Natal, Durban 4001, South Africa; shakeelkader2006@gmail.com; 4 Division of Research Capacity Development, South African Medical Research Council (SAMRC), Tygerberg, Cape Town 7505, South Africa; 5 SAMRC Precision Oncology Research Unit (PORU), DSTI/NRF SARChI Chair in Precision Oncology and Cancer Prevention (POCP), Pan Africa Cancer Research Institute (PACRI), University of Pretoria, Hatfield 0028, South Africa; 6 Biomedical Sciences Department; School of Life and Consumer Sciences, College of Agriculture and Environmental Sciences, University of South Africa, Florida Campus, Johannesburg 1710, South Africa

**Keywords:** HIV-helminth co-infection, immune checkpoint molecules, immunosuppressive microenvironment, CD28 downregulation, Th2-type immunity, PD-1 and TIM-3, chronic infections and cancer, regulatory T cells (Tregs), antitumor immunity dysfunction

## Abstract

South Africa has the highest HIV prevalence globally, often co-occurring with helminth infections in impoverished regions. The coexistence of these infections leads to immunological interactions, potentially enhancing oncogenesis by upregulating immune checkpoint molecules (ICs) among other effects. Notably, most ICs are overexpressed in cancer and correlated with its progression. Helminth infections trigger Th2-type immunity, increasing immunosuppressive M2 macrophages, regulatory T cells, and associated IC molecules. PD-L2 is reported to contribute to Th2-type immunity induced by helminth infections. Similarly, TIM-3, elevated during chronic viral infections, induces a similar immunosuppressive profile. CTLA-4 and PD-1 impact T-cell function by interacting with CD28, crucial for T-cell function. CD28 is downregulated in chronic infections and cancer. This study investigated the impact of HIV-helminth co-infection on co-stimulatory and co-inhibitory molecule profiles associated with antitumor immunity. Using 78 serum samples collected from March 2020 to May 2021, participants were categorized into uninfected control (no HIV and helminth infections), HIV-infected, helminth-infected, and HIV-helminth co-infected groups. Multiplex immune regulatory molecule assay analysis was conducted. The data were analyzed using multivariate regression analysis and adjusted for confounders (age, gender, BMI, ART, supplements, and other chronic diseases). The uninfected control group was used as the baseline reference group for analysis. HIV-infected individuals had higher PD-1 (adjusted β = 0.12, *p* = 0.034) and TIM-3 (adjusted β = 23.15, *p* = 0.052) levels, with the latter showing a trend toward significance. However, lower CD28 levels (adjusted β = −651.95, *p* = 0.010) were observed. Helminth-infected individuals had higher TIM-3 levels (adjusted β = 20.98, *p* = 0.020). The co-infected group had higher PD-1 (unadjusted β = 0.18, *p* = 0.0046) and PD-L2 (adjusted β = 7.95, *p* = 0.033) levels. A significant decrease in CD28 profile was observed across all infected groups: HIV-infected (adjusted β = −651.95, *p* = 0.010), helminth-infected (adjusted β = −674.32, *p* = 0.001), and co-infected (adjusted β = −671.55, *p* = 0.044). The results suggest that HIV-helminth co-infections alter immune checkpoint markers, potentially increasing cancer risk by promoting an immunosuppressive microenvironment that hinders anti-cancer immunity. CD28’s downregulation underscores immune inefficiency in chronic diseases. Addressing these co-infections is crucial for improving HIV care and potentially reducing cancer risks through targeted strategies.

## 1. Introduction

People living with human immunodeficiency virus (HIV) are particularly susceptible to various infections [1], including helminths, which are common in children and adults residing in poorly resourced regions of low-middle-income countries (LMICs) [2,3,4]. The immunological and biological interaction between HIV and helminth infections is complex, and it remains unclear whether helminth infections definitely increase susceptibility to HIV and pathogenesis or vice versa [5]. Certain helminth-induced immunological phenotypes might suggest that helminths increase susceptibility to HIV [6]. Helminth infections have been shown to have negative effects on HIV immune responses, predominantly modulating type 2 T helper (Th2) cell responses. Mulu et al. (2015) noted that 12 weeks after deworming and antiretroviral therapy (ART), there was a significant reduction in serum IgE levels among symptomatic HIV-helminth co-infected individuals, indicating a downregulation of the Th2 immune response [7]. The ability to polarize Th1 into Th2 immunity at the early stages of an insult is what helminths have in common with cancer. Secretory molecules from helminths can also recruit immunosuppressive and cancer-favoring regulatory T cells (Tregs), which aid in the sustenance and survival of chronic infections [8] and cancer cells [9].

A systematic review by Alghanmi et al. (2024) [10] demonstrated that helminth-derived proteins can influence the host’s immune response, serving as an adaptive strategy for helminth survival. In mice models of colitis, the administration of these proteins was found to suppress the production of Th1/Th17 cytokines, such as IL-1β, IL-6, TNF-α, IL-17, and IFN-γ, while enhancing the levels of Th2/Treg cytokines, including IL-4, IL-13, TGFβ, and IL-10, in serum, colon tissue, and spleen samples [10]. Helminth infections induce the recruitment of immune cells, such as eosinophils and basophils, to sites of helminth infection which is mediated by Th2 cytokines. This is an attempt to expel the invading worms. However, the helminths have developed mechanisms to evade the immune attack, and eventually a modified Th2 response ensues, to facilitate the parasite’s survival [11,12]. The modified Th2 response is accompanied by the production of alternatively activated macrophages (AMMs or M2) among others. Th2 response assists in controlling excessive inflammation, thereby creating a more hospitable environment for the helminths to persist within the host [13].

Helminth-derived vesicles also have a major contribution to immune regulation. Their intracellular effect is mostly achieved through helminth-derived products (HDPs), which interact with toll-like receptors (TLRs) in antigen-presenting cells (APCs), not only modulating TLR expression, but also skillfully influencing their intracellular signaling pathways. Certain HDPs can redirect TLR4 signaling toward the MAPK pathway and ERK1/2 activation, thereby promoting the induction of Treg and Th2 responses [14,15]. Although not well understood, helminth infections have been shown to promote myeloid-derived suppressor cells (MDSCs) and alternatively activated macrophages (AAMs), also known as M2 macrophage-mediated immunosuppression [16], Table 1. Furthermore, helminths induce increased numbers of M2 macrophages, that, among other functions of driving the host defense, prevent type 2 immunopathology. They mitigate and repair parasite-induced tissue damage and generate an anti-inflammatory environment through the production of anti-inflammatory mediators such as interleukin 10 or prostaglandin E2 which assist the parasite to evade host immunity [17,18].

The co-stimulatory molecule, CD28, plays a significant role in generating, activating, and regulating antigen-specific T cells [19,20]. However, helminths and HIV have been shown to decrease the cellular expression of CD28 while increasing the expression of immune checkpoints, such as cytotoxic T-lymphocyte Antigen-4 (CTLA-4) and programmed cell death 1 (PD-1), aiding in the reduction of T cell activation and proliferation [21]. In addition, Tregs and M2 macrophages express co-inhibitory receptors CTLA-4, the lymphocyte activation gene-3 (LAG3), the T cell immunoglobulin and mucin domain-containing molecule 3 (TIM-3) [22], and PD-1 and its ligands, PD-L1/2 [23] (Table 1). Helminth and other parasitic infections can induce the expression of PD-L2 from activated macrophages, which bind to PD-1 on activated T cells, leading to anergy and reduced activation [24]. The expression of PD-L2 on peritoneal macrophages has been linked to increased susceptibility to *Fasciola hepatica* infection, as demonstrated in PD-L2 deficient mice. The authors found that PD-L2 expression is crucial for M2 macrophage polarization during helminth infections and is necessary to maintain the balance between Th1- and Th2-type immune responses [24]. Another related immune-inhibitory molecule, with similar functions to both PD-1 and CTLA-4, is the B and T lymphocyte attenuator (BTLA) receptor [25]. An infection with the helminth *Strongyloides ratti* has been shown to upregulate the BTLA pathway. Conversely, reducing BTLA or its ligand, the herpes virus entry mediator (HVEM), impairs the helminth’s ability to evade the host’s immune responses, thus compromising its survival [26]. The helminth-induced upregulation of immune checkpoints and reduction of costimulatory molecules, such as CD28 (as shown in Table 1), share similarities in immune modulation observed in cancer with a potential significant impact on cancer immunotherapy. Dual infection with helminths and HIV may further amplify immunosuppressive effects, potentially creating a favorable environment for cancer development and progression.

**Table 1:** Shared immunosuppressive mechanisms between helminths and cancer.

Mechanism	Helminths	Cancer	References
PD-1/PD-L1 Upregulation	Helminths upregulate PD-1 and PD-L1 on T cells and antigen-presenting cells, leading to immune suppression.	Tumors express PD-L1 to evade immune detection.	[24,27]
Treg Expansion and Th2 Skewing	Helminths induce Treg expansion and shift immune responses from Th1 to Th2, dampening pro-inflammatory responses.	Tumors recruit Tregs to suppress anti-tumor immunity.	[28,29]
MDSC and M2 Macrophage Induction	Helminths promote MDSC accumulation and M2 macrophage polarization, which suppress immune responses.	Tumors recruit MDSCs and polarize macrophages to M2 phenotype, aiding tumor progression.	[16,30]
Secretion of Immunomodulatory Molecules (IL-10, TGF-β)	Helminths secrete IL-10 and TGF-β, leading to immune suppression and tissue remodeling.	Tumors secrete IL-10 and TGF-β to create an immunosuppressive microenvironment.	[31,32]
CTLA-4 Expression	CTLA-4 expression in helminth-infected individuals, suggesting a role in immune regulation might be mainly from immunosuppressive cells such as Tregs.	Tumors exploit CTLA-4 checkpoints to suppress T-cell activation.	[27,32,33]

Programmed cell death protein 1 (PD-1), Programmed cell death ligand 1 (PD-L1), T helper (Th), Myeloid-derived suppressor cells (MDSC), M2 macrophages (M2), Interleukin (IL), Transforming growth factor-beta (TGF-β), Cytotoxic T-lymphocyte-associated protein 4 (CTLA-4).

The benefits of blocking the expression of ICs such as CTLA-4 and PD, along with their ligands (CD80/86 and PD-L1/2, respectively) in cancer [34] have been extensively investigated. Given the profound immunomodulatory effects of helminths and HIV co-infection, it is imperative to investigate the potential risks these co-infections may pose in cancer initiation and development. Understanding these interactions can aid in the development of comprehensive treatment and prevention strategies that address the dual burden of these infections and their possible role in cancer pathogenesis. This study, therefore, aimed to investigate the association between HIV-helminth co-infection and the expression of co-stimulatory and co-inhibitory molecules involved in immunoregulatory pathways that influence antitumor immune responses.

## 2. Materials and Methods

### 2.1. Study Design

This was a cross-sectional observational sub-study based on the main project with ethics approval number BE351/19. A separate approval (BREC/00005458/2023) for this study was received from the Biomedical Research Ethics Committee of the University of KwaZulu Natal (UKZN), Durban, South Africa.

### 2.2. Study Population and Sampling

The present sub-study used stored samples collected for the main project conducted in a peri-urban area south of Durban, KwaZulu-Natal, South Africa, from March 2020 to May 2021. Whole blood and stool samples were collected from consenting participants for the master study and utilized in the retrospective cohort. The recruitment of participants has been described in detail elsewhere [3]. A total of 78 participants were purposively selected based on their infection status. Those with complete datasets for additional analyses were then divided into four groups [HIV and helminth uninfected control (*n* = 20), HIV infected (*n* = 20), helminth infected (*n* = 20), and HIV-helminth co-infected (*n* = 18)]. Every second participant was selected from each pool in the respective group until there were 20 participants per group. Only 18 were HIV and helminth co-infected; thus, by default, this group comprised 18 participants.

### 2.3. Parasite Detection

Collected stool samples were processed using the Kato–Katz technique for detecting intestinal helminths. A modified fecal parasite concentrator technique was performed using a mini Parasep^®^ SF stool concentrator kit to concentrate potential parasite ova and cysts for increased detection sensitivity. To further enhance the sensitivity of coproscopy, serum samples were tested for the presence of Ascaris lumbricoides-specific IgE (>0.35 kU/L) and IgG4 (>0.15 kU/L) antibodies using a Phadia™ 200 instrument (Phadia AB, Thermo Fisher Scientific, Uppsala, Sweden). This method is described in more detail elsewhere [3].

### 2.4. Full Blood Cell Count and Detection of HIV Status

Serological HIV testing of the participants utilized the Alere Determine^TM^ HIV-1/2 Ag/Ab Combo rapid test, manufactured by (Abbott Diagnostics Scarborough, Inc., Scarborough, ME, USA), with inconclusive results confirmed using the ICT HIV-1/2 Ag/Ab test kit (ICT diagnostics, Cape Town, South Africa). Whole blood was analyzed using the full blood count analyzer (AQUIOS CL Flow Cytometer System, Beckman Coulter Life Sciences, Brea, CA, USA) to determine differential white blood cell counts. A previous publication provides a more detailed description of the methods [35].

### 2.5. Analysis of Immune Regulatory Molecules

Serum stored at −80 °C was thawed at 4–8 °C overnight. From a human ProcartaPlex immunoassay kit (Invitrogen, Thermo Fisher Scientific, MA, USA), we analyzed CD27, CD28, CD137 [4-1BB], BTLA, CTLA, CD80, IDO, LAG-3, PD-1, PD-L2, and TIM-3 immunoregulatory profiles. The experimental procedure, standard samples, magnetic beads, and patient samples were prepared according to the manufacturer’s instructions. Upon the addition of diluted serum samples (1:2) and standards, an antibody specifically directed against each target is covalently coupled to color-coded polystyrene beads, which react with the serum samples containing unknown analyte concentrations or with standard solutions of known concentrations. After a washing step to remove unbound proteins, a biotinylated detection antibody specific to a different epitope on the analyte is added to form a sandwich of antibodies around the analyte. Thereafter, a biotinylated detection antibody is added, followed by another washing step. Streptavidin-R-Phycoerythrin is added to bind to the detection antibody. The concentration of each analyte in a single well was read on a Bio-Plex^®^ 200 System (Bio-Rad Laboratories, Inc., Hercules, CA, USA) and the concentration of the target analyte was calculated by Bio-Plex Manager™ 4.0 software using a standard curve derived from the supplied recombinant standard; the results are reported in pg/mL.

### 2.6. Statistical Analysis

Multivariate regression analysis was performed on the IC profiles. The results are reported as unstandardized beta coefficients (β) along with their 95% confidence intervals (CIs). In Table 2, for each IC marker, data in row “A” represent the unadjusted data, while data in row “B” represent data adjusted for age, gender, BMI, vitamin and nutrient supplement intake, antiretroviral treatment, and chronic illnesses. A *p*-value < 0.05 was considered statistically significant. In this analysis, the uninfected control group served as the baseline reference for comparison. Statistical analysis was conducted with Stata Statistical Software: Release 17 (StataCorp LLC, College Station, TX, USA, 2019).

## 3. Results

### 3.1. Patient Demographics

The demographics of study participants are shown in Table 2. Study participants (*n* = 78) were divided into four groups: the uninfected control (or no HIV or helminth infection) (*n* = 20), HIV infection only (*n* = 20), helminth infected only (*n* = 20), and HIV-helminth co-infections (*n* = 18).

The average age of participants across the groups was as follows: Controls had a mean age of 46.4 years (±17.3); HIV-infected only had a mean age of 42.9 years (±12.3); Helminth-infected only had a mean age of 37.1 years (±16.4); and those with HIV and helminth co-infection had a mean age of 39.4 years (±10.0). The differences in age between these groups were not statistically significant (*p*-value = 0.200). Most participants were female, and there was no significant difference in gender distribution between the groups. A slight variation in BMI was observed, with the helminth-infected only group having the highest mean BMI (29.6 ± 8.3 kg/m²) and the HIV and helminth co-infection group having the lowest (27.0 ± 5.4 kg/m²). Notably, the CD4 count was significantly lower in the HIV-infected only (629 ± 448 u/L) and HIV and helminth co-infection (579 ± 370 u/L) groups compared to the other groups, with a *p*-value of 0.001. Regarding the CD4/CD8 ratio, the HIV-infected (0.75 ± 0.44) and HIV-helminth co-infected (0.90 ± 0.62) groups had significantly lower ratios compared to the uninfected control group. Furthermore, the helminth-infected (1.43 ± 0.60) group had significantly higher ratio values compared to the HIV group (0.75 ± 0.44). The white cell and neutrophil counts were slightly lower in the HIV-infected only and HIV and helminth co-infection groups. This trend is consistent with the lower lymphocyte counts observed in the HIV and helminth co-infection group compared to other groups. Additionally, a slight increase in monocyte counts was observed in the helminth-infected only group compared to the other groups.

### 3.2. The Prevalence of Parasites

**Figure 1:**
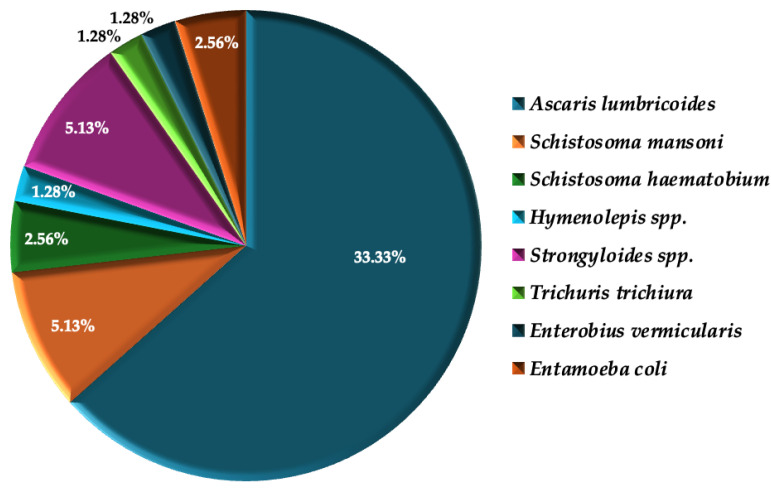
The prevalence of species-specific helminth infections [*n* (%)].

Figure 1 below indicates the prevalence of helminth infections in the study population.

*Ascaris lumbricoides* was the most prevalent parasitic infection (*n* = 26, 33.33%). Other helminth species identified included *Strongyloides species* (*n* = 4, 5.13%), *Schistosoma mansoni* (*n* = 4, 5.13%), and *Schistosoma haematobium* (*n* = 2, 2.56%). Additionally, *Hymenolepis species****,*** *Trichuris trichiura,* and *Enterobius vermicularis* were each detected (*n* = 1, 1.28% each). Notably, some individuals harbored multiple species, such as two participants co-infected with *Ascaris lumbricoides* and *Entamoeba coli*. However, infections with *Entamoeba coli* alone were excluded from the analysis. Percentages are based on the number of participants infected with each species, not the total number of infections, as some individuals harbored multiple species.

### 3.3. The Profile of Immune Checkpoints/Co-Inhibitory Molecules, Immune Co-Stimulatory Molecules, and Immunosuppressive Enzymes

The association of immune regulatory molecule profiles during HIV and helminth single infection and co-infection is presented in Table 3. Data were adjusted for age, gender, BMI, nutrient supplement intake, ARVs, and chronic illnesses.

The study observed a significant increase in PD-1 levels in HIV-infected (adjusted β = 0.12, *p* = 0.034) and co-infected (unadjusted β = 0.18, *p* = 0.046) individuals. A significant increase in PD-L2 expression was observed between the uninfected control and co-infected groups (unadjusted β = 7.95, *p*= 0.033). A marginally significant increase in the expression of TIM-3 was noted in the HIV singly infected group (adjusted β = 23.15, *p* = 0.052) and a significant increase was detected in the helminth singly infected group (adjusted β = 20.98, *p* = 0.020). The expression levels of CD28 were decreased across all groups: HIV-infected (adjusted β = -651.95, *p* = 0.010) (unadjusted β = -594.33, *p* = 0.002); helminth-infected (adjusted β = -674.32, *p* = 0.001) (unadjusted β = -590.42, *p* = 0.001), and co-infected (adjusted β = -671.55, *p* = 0.044) (unadjusted β = -594.01, *p* = 0.003) groups.

## 4. Discussion

The present study aimed to explore the impact of HIV-helminth co-infection on immune checkpoint molecules. Data were adjusted for variables such as age, gender, BMI, ART status, supplement use, and other chronic diseases. The results show distinct alterations in immune checkpoint profiles among the groups. HIV-infected and co-infected individuals had significantly increased levels of PD-1. TIM-3 expression was marginally increased in HIV-infected individuals and significantly increased in the helminth-infected group. Notably, CD28 expression was significantly decreased across all infected groups. The observed downregulation of CD28 suggests a reduced T cell co-stimulatory capacity, which is associated with impaired immune responses in chronic infections. This reduction in CD28 expression could also be due to immunosenescence, further contributing to an immunosuppressive environment that facilitates cancer initiation. Taken together, these findings suggest that HIV-helminth co-infection modulates immune checkpoint markers, potentially fostering an immunosuppressive environment that may impair anti-cancer immunity and increase the risk of cancer.

### 4.1. CD28 Dysregulation and PD-1 Expression as Potential Pathways to Oncogenesis

In this study, the expression levels of CD28 were significantly downregulated across all groups: HIV-infected [A (*p* = 0.002); B (0.010)], helminth-infected [A (*p* = 0.001); B (*p* = 0.001)], and HIV-helminth co-infected [A (*p* = 0.044); B (*p* = 0.003)] in both adjusted and unadjusted data. A study showed that a decrease in CD28 + CD8 + cells in helminth-infected individuals normalized after anti-helminth treatment compared to the untreated infected individuals [36]. Ndlovu (2013) highlighted that CD28 is necessary for the induction of Th2 immunity against the helminth, *Nippostrongylus brasiliensis*, associated with impaired memory CD4⁺ T cell responses [37]. CD28 is crucial not only for anti-tumor T cell proliferation but also for developing and maintaining Tregs [38]. Cellular senescence arises in cells with irreversible damage, halting their ability to divide and often triggering the persistent release of pro-inflammatory molecules, a phenomenon known as the senescence-associated secretory phenotype. In T cells, the downregulation of CD28 has been closely linked to the onset of cellular senescence, contributing to their diminished functionality and impaired immune responses [39,40]. CTLA-4 is an inhibitory molecule that competes with the co-stimulatory molecule CD28 for binding, thereby regulating T-cell responses against pathogens and cancer [41]. Blocking CTLA-4 in helminths (*Nippostrongylus brasiliensis*) improved CD28 T cell activation, resulting in clearance of the parasite and normalization of immune responses [42]. In this study, CTLA-4 levels were increased in the HIV-infected, helminth-infected, and HIV-helminth co-infected groups compared to the uninfected control group; however, the differences were not statistically significant.

Studies have shown that PD-1 uses CD28 post-TCR activation to block immune responses, although PD-1 does not entirely depend on CD28 for immunosuppression [43]. In the current study, a significant increase in PD-1 expression (a cancer-favoring phenomenon) was observed in HIV individuals (*p* = 0.034) compared to controls. Blocking the PD-1/PD-L1 pathway has been shown to restore the activity of exhausted virus-specific CD8 + T cells, resulting in regulated chronic HIV infection [44] and enhanced anti-cancer immunity [45]. The increase in PD-1 levels observed in the current study correlates with a decrease in CD28 in the HIV singly infected group, which potentially contributes to immunosuppression and susceptibility to cancer initiation. Furthermore, Vivar and colleagues reported that T cells lacking CD28 expression in HIV patients were found to be prone to apoptosis and exhibited a low proliferation rate compared to HIV patients on ARVs who had a controlled chronic viral infection [46]. This is exacerbated by HIV’s ability to reduce or impair CD28 + T cells [47]. However, while polymorphisms in the CD28 co-signaling molecule have been shown to contribute to cancer initiation and progression, significant work remains to fully develop its potential as both a screening/diagnostic tool and a therapeutic marker [48].

### 4.2. PD-L2 Expression in a HIV-Helminth Co-Infected Group and Its Potential Role in Oncogenesis

The current study found a significant increase in PD-1 ligand (PD-L2) expression in the helminth-HIV infection (*p* = 0.033) group. Imai et al. (2023) found the expression of PD-L2 to be higher in metastatic cancer cells (47%) compared to PD-L1 (11%), with no PD-L1 expression observed in tumor-infiltrating lymphocytes (TILs) (0%), whereas 64% of TILs expressed PD-L2 [49]. Yuki et al. reported different findings, showing PD-L2 expression in cancer tumors as 0% and in associated lymphocytes as 20.3%, in contrast to PD-L1, which was expressed at 0.8% in tumors and 46.9% in lymphocytes [50]. Despite conflicting findings, these studies emphasize the importance of this underexplored immune checkpoint in cancer and call for further research to clarify its role in oncogenesis. Similarly, while helminth infections can induce PD-L2 expression in macrophages [51], additional research is required to fully understand the role of the PD1-PD-L1/2 axis in oncogenesis before considering it as a potential prognostic biomarker.

### 4.3. TIM-3 Increase and Its Implications for Cancer Onset in HIV- and Helminth-Infected Groups

A significant increase in TIM-3 levels, a molecule known for its role in cancer initiation, was observed in both the HIV-infected (marginally significant *p* = 0.052) and helminth-infected (*p* = 0.020) groups compared to the controls. Prévost et al. report that the HIV-1 protein Vpu decreases the surface expression of TIM-3 on infected CD4 + T cells, indicating that TIM-3 may contribute to suppressing viral replication. By downregulating TIM-3, Vpu counteracts this inhibitory effect, thereby facilitating increased viral replication and spread [52]. However, changes in TIM-3 levels during parasitic infections appear to be cell-specific. Hou et al. reported that *Plasmodium falciparum* downregulated TIM-3 expression in the circulating monocytes of infected patients, with an even more pronounced effect in splenic macrophages in mice studies. Blocking TIM-3 enhanced monocyte/macrophage activity, aiding in parasite clearance [53]. Similarly, Hou et al. observed differential TIM-3 expression in immune cells across various organs in mice infected with *Schistosoma japonicum*. The study found a higher infiltration of immune cells, CD4/8 T cells, natural killer (NK) cells, and monocytes in the liver with lower TIM-3 levels in CD8 T cells and monocytes in the spleen. Helminth infection upregulated TIM-3, leading to poor NK cell functionality. Notably, blocking TIM-3 improved the NK cell function and inhibited cancer-favoring M2 macrophages, also known as tumor-associated macrophages (TAMs) [54]. Additionally, increased TIM-3 expression in the tumor microenvironment is associated with a higher metastatic stage (*p* < 0.05) and decreased overall survival [55]. Thus, taken together, TIM-3 might potentially serve as a screening marker for cancer risk in HIV-helminth co-infected patients.

### 4.4. Implications for Colorectal Cancer (CRC) Risk

**Figure 2:**
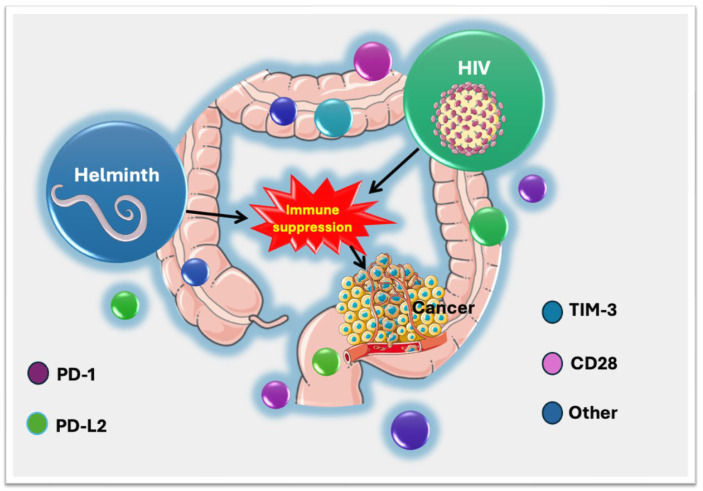
Implications of HIV and helminth co-infection in altering immune checkpoint profiles and subsequent CRC risk. The gastrointestinal mucosa is the primary site for HIV and parasitic infections because (i) HIV infection and immune response establishment begin in the gut-associated lymphoid tissues (GALTs), and (ii) intestinal parasites reside in the same environment, which is also the site of CRC origin. HIV-helminth infections promote the immuno-suppressive microenvironment via the secretion of molecules that promote anti-tumor immune responses including immune checkpoints PD-1/PD-L2 and TIM-3. Downregulation of co-stimulatory molecules such as CD28 is an attempt to prevent T cell activation and evade immunological attack. Other than chronic inflammation induced by helminths, other molecules/cells, generally known to be favored by both helminths and cancer cells increase the chances of individuals with HIV-co-infections developing CRC.

The gastrointestinal mucosa plays a very central role in HIV and parasitic infections because (i) early HIV initial replication and immune response establishment start in the gut-associated lymphoid tissues (GALTs) [56], and (ii) intestinal parasites reside in the same environment, establishing chronic low-grade inflammation while modulating the immune response. Coincidentally, this is also the same anatomical site for colorectal cancer (CRC) origin. It is therefore biologically plausible that these immunological interactions (Figure 2) accentuate the risk of cancer development in the colon relative to other cancers.

Helminth infections are known for promoting intestinal Th2-mediated inflammation through suppression of Th1 responses, causing cellular damage and an increased risk of co-infections [57]. A previously reported study in the same cohort found HIV-helminth co-infection is associated with elevated pro-inflammatory immune responses, which indicates that helminths have a deleterious effect on HIV immune responses [28]. Both HIV [58] and parasite [27] single infections are associated with dysregulated immune checkpoint profiles, with no reported studies on co-infected individuals. These alterations suggest a dysregulated immune response that could contribute to chronic inflammation, a known factor in the initiation of CRC. Helminth immune dysregulation is implicated in CRC initiation [59]. Intestinal helminth infections have been reported to drive carcinogenesis in colitis-associated colon cancer [59]. Helminth also promotes microbial dysbiosis [60], which is associated with oncogenesis and CRC risk [61].

As mentioned, in the present study, TIM-3 expression was elevated in the HIV and helminth singly infected groups, PD-1 and PD-L2 expression was elevated in the co-infected group, and CD28 was lower in all study groups. Elevated TIM-3 and PD-1 expression in CRC tumors was associated with a higher metastatic stage and a decline in overall survival rate [62]. Elevated PD-1 [44] and decreased CD28 levels in [62] HIV-infected individuals contribute to immunosuppression and susceptibility to CRC initiation. Helminth infections play a role in inducing PD-L2 expression in macrophages [51]. Significantly higher PD-L2, and not PD-L1, expression was reported in metastatic CRC cells [63]. Therefore, populations affected by both HIV and helminth infections may face a heightened risk of CRC compared to those without such co-infections.

## 5. Challenges and Limitations

The sample size for this study may limit the statistical power to detect subtle differences between the groups, and a larger size might increase the robustness of the results and improve generalizability. The cross-sectional design further restricts the ability to infer causality between HIV-helminth co-infection and the immune profiles assessed. Longitudinal studies would be needed to track changes over time and better understand the temporal dynamics and causality of these immune alterations. Another limitation of the study is the unknown duration of both HIV and helminth infections since the acute and chronic phases of both infections are accompanied by distinct immunological changes as the diseases progress from acute to chronic [21]. Likewise, since the implementation of the “test and treat” HIV regulation in South Africa in 2016, the duration of HIV treatment is also unknown [64,65]. Finally, while the study focused on co-stimulatory and co-inhibitory molecules, other immune markers known to play a role in modulating antitumor immunity should be explored in future research.

## 6. Conclusions

This study’s findings suggest that HIV and helminth co-infections impact immune checkpoint signaling, particularly through the reduction of CD 28 expression, potentially increasing cancer risk due to immune response inefficiency. The study highlights that immune homeostasis, which is important for eliminating cancer cells and pathogens, is disrupted under these co-infection conditions. The changes in immune checkpoint markers observed in HIV-helminth co-infected individuals may elevate the risk of developing cancer. Several negative immunoregulators, such as CTLA4, PD1, and its ligands, modulate the immune response to maintain homeostasis and prevent hyperactivation or autoimmunity. Blocking these T cell immune checkpoint molecules has revolutionized immunotherapy, significantly advancing treatment for several cancers [66]. Co-infections of HIV and helminths have been shown to alter immune checkpoint signaling pathways by regulating markers such as PD-1, PD-L1/2, [67] and TIM-3 [68]. These alterations suggest a dysregulated immune response that could contribute to chronic inflammation, a known factor in the initiation of cancer. Chronic inflammation, associated with immune dysregulation, is implicated as a potential mechanism linking these infections to cancer initiation [69]. Therefore, populations affected by both HIV and helminth infections may face a heightened risk of cancer compared to those without such co-infections. Addressing HIV-helminth co-infections through comprehensive treatment and prevention strategies is crucial. Effective management of these infections not only improves HIV outcomes but also potentially mitigates the associated risk of developing cancer [70,71]. Integrating parasitic infection control measures into HIV care programs is essential, especially in regions where both diseases are prevalent [72]. This study underscores the broader public health implications of HIV and helminth co-infections, particularly in regions with high prevalence rates. By addressing these co-infections, healthcare systems can enhance overall health outcomes and potentially reduce the burden of cancer within these populations. Lastly, this study advocates for integrated approaches in healthcare policies and practices to effectively manage co-infections and improve long-term health outcomes in affected communities.

## Figures and Tables

**Table 2:** Participants’ demographics and blood cell counts (*n* = 78).

Parameters	Uninfected Controls (*n* = 20)	HIV-Infected Only (*n* = 20)	Helminth-Infected Only (*n* = 20)	HIV + Helminth Co-Infection (*n* = 18)	*p*-Value
Age (years)	46.4 ± 17.3	42.9 ± 12.3	37.1 ± 16.4	39.4 ± 10.0	0.200
Gender, *n* (%)					
Males	6 (30)	9 (45)	7 (35)	6 (30)	0.725
Females	14 (70)	11 (55)	13 (65)	14 (70)	
BMI (kg/m^2^)	28.0 ± 6.9	26.0 ± 7.0	29.6 ± 8.3	27.0 ± 5.4	0.418
CD4 count (u/L)	996 ± 375 *	629 ± 448 ^#^	892 ± 197 *	579 ± 370 ^#^	0.001
CD8 (u/L)	781 ± 341	910 ± 455	724 ± 299	742 ± 354	0.3801
CD4/CD8 ratio	1.57 ± 0.92 *	0.75 ± 0.44 ^$^	1.43 ± 0.60 ^#^	0.90 ± 0.62 ^#,$^	0.0004
Viral load (copies/mL)					
*n* (%)					
<20		13 (65)		11 (61)	1.000
>20		7 (35)		7 (39)	
White cell count (×10^9^/L)	6.52 ± 1.96	5.65 ± 2.06	6.07 ± 1.83	5.16 ± 1.81	0.163
Neutrophils (×10^9^/L)	3.61 ± 1.50	3.05 ± 1.41	3.19 ± 1.34	2.65 ± 1.04	0.180
Lymphocytes (×10^9^/L)	2.27 ± 0.61	2.02 ± 1.00	2.21 ± 0.50	1.79 ± 0.66	0.164
Monocytes (×10^9^/L)	0.42 ± 0.12	0.40 ± 0.12	0.47 ± 0.15	0.39 ± 0.11	0.206

The different superscript values for a given parameter between the different groups denote statistical significance.

**Table 3:** Multivariate association of immune regulatory molecule profiles during HIV and helminth single infection and co-infection.

	Unstandardized β–Coefficient Values (Reference Group: Uninfected Controls)						
Parameters		HIV Infected		Helminth Infected		HIV and Helminth Co-Infected	
		β (95% CI)	*p*	β (95% CI)	*p*	β (95% CI)	*p*
Immune Checkpoints/Co-Inhibitory Molecules							
Programmed cell death (PD)-1	A	0.05 (−0.04–0.14)	0.251	0.13 (−0.11–0.37)	0.266	0.18 (0.00–0.35)	0.046
	B	0.12 (0.01–0.23)	0.034	0.15 (−0.16–0.45)	0.335	0.19 (−0.13–0.51)	0.235
PD-ligand (L)2	A	0.57 (−12.41–13.54)	0.929	6.29 (−9.95–22.52)	0.436	7.95 (0.67–15.23)	0.033
	B	7.68 (−9.11–24.480	0.351	12.53 (−8.38–33.45)	0.228	6.96 (−3.73–17.66)	0.193
B and T lymphocyte attenuator (BTLA)	A	0.79 (−1.24–2.83)	0.431	2.26 (−1.70–6.22)	0.253	15.15 (−17.03–47.33)	0.342
	B	1.96 (−0.51–4.42)	0.114	3.66 (−1.07–8.39)	0.124	44.05 (−10.47–98.57)	0.107
Cytotoxic T-lymphocyte Antigen-4 (CTLA-4)	A	4,03 (−20.65–28.71)	0.743	4.59 (−16.74–25.93)	0.665	12.03 (−33.70–57.77)	0.597
	B	3.99 (−31.11–39.09)	0.813	5.05 (−15.93–26.04)	0.626	33.97 (−37.38–105.33)	0.339
T cell immunoglobulin and mucin domain containing molecule 3 (TIM-3)	A	13.58 (−5.73–32.90)	0.161	6.02 (−10.38–22.43)	0.459	12.58 (−8.68–33.84)	0.235
	B	23.15 (−0.20–46.50)	0.052	20.98 (3.52–38.45)	0.020	18.11 (−13.02–49.25)	0.238
Immunosuppressive Enzymes							
Indoleamine 2,3-dioxygenase (IDO)	A	−9.47 (−31.92–12.98)	0.394	−5.84 (−27.65–15.97)	0.589	−10.39 (−32.94–12.16)	0.352
	B	−9.44 (−39.65–20.77)	0.522	−2.40 (−26.65–21.84)	0.840	−6.64 (−47.18–33.89)	0.735
Immune Co-Stimulatory Molecules							
CD27	A	803.69 (−310.51–1917.89)	0.150	−23.60 (−544.54–497.34)	0.927	213.92 (−403.73–831.57)	0.483
	B	911.70 (−535.26–2358.66)	0.204	427.14 (−156.24–1010.53)	0.144	511.32 (−349.16–1371.80)	0.229
CD28	A	−594.33 (−946.57–−242.10)	0.002	−590.42 (−902.25–−278.59)	0.001	−594.01 (−958.93–−229.08)	0.003
	B	−651.95 (−1126.74–−177.15)	0.010	−674.32 (−1054.48–−294.16)	0.001	−671.55 (−1322.53–−20.58)	0.044
CD80	A	−0.93 (−2.44–0.59)	0.220	0.81 (−2.58–4.21)	0.629	2.32 (−2.84–7.48)	0.363
	B	−0.86 (−2.94–1.22)	0.398	1.06 (−3.15–5.28)	0.608	5.92 (−3.42–15.26)	0.200
CD137	A	0.27 (−0.14–0.689)	0.190	1.40 (−4.63–3.26)	0.136	1.18 (−0.14–2.50)	0.077
	B	0.51 (−0.43–1.06)	0.069	1.48 (−0.94–3.90)	0.219	2.31 (−0.129–4.74)	0.062

The uninfected control group (study participants with no HIV and no helminth infections) was the reference group for multivariate analysis. A: Unadjusted data. B: Data were adjusted for age, gender, BMI, vitamin and nutrient supplements intake, antiretroviral treatment, and chronic illnesses.

## Data Availability

Data generated from this study can be accessed from the corresponding author upon reasonable request.
